# Relationship between daily physical activity and aerobic fitness in adults with cystic fibrosis

**DOI:** 10.1186/s12890-015-0036-9

**Published:** 2015-05-09

**Authors:** Daniela Savi, Marcello Di Paolo, Nicholas Simmonds, Paolo Onorati, Mattia Internullo, Serena Quattrucci, Banya Winston, Pierantonio Laveneziana, Paolo Palange

**Affiliations:** Department of Pediatrics and Pediatric Neurology, Cystic Fibrosis Center, Sapienza University of Rome, 00185 Rome, Italy; Department of Public Health and Infectious Diseases, Sapienza University of Rome, 00185 Rome, Italy; Department of Cystic Fibrosis, Royal Brompton Hospital and Imperial College, SW3 6NP, London, UK; NIHR Respiratory BRU, Royal Brompton Hospital NHS Foundation Trust, SW3 6NP, London, UK; Neurophysiologie Respiratoire Expérimentale et Clinique, Sorbonne Universités, UPMC University Paris 06, UMR_S, 1158 75005 Paris, France; Neurophysiologie Respiratoire Expérimentale et Clinique, INSERM, UMR_S, 1158 75005 Paris, France; AP-HP, Groupe Hospitalier Pitié-Salpêtrière Charles Foix, Service des Explorations Fonctionnelles de la Respiration, de l’Exercice et de la Dyspnée, 75013 Paris, France; Eleonora Lorrillard-Spencer Cenci Foundation, Rome, Italy

**Keywords:** Daily physical activity, Lactic threshold, Exercise fitness

## Abstract

**Background:**

The best clinical practice to investigate aerobic fitness includes measurements obtained during cardiopulmonary exercise testing (CPET), however it remains an underutilised clinical measure in cystic fibrosis (CF). To investigate this further, different methods of quantifying exercise capacity in CF are required. The possibility that measuring physical activity (PA) by a portable accelerometer could be used to assess the CF aerobic state and could be added among the CPET surrogates has not been investigated. The aim of this study was to examine the relationship between PA and exercise fitness both at submaximal and maximal levels in clinically stable adults with CF.

**Methods:**

Thirty CF patients (FEV_1_ 71 ± 19% predicted) and fifteen healthy controls undertook an incremental CPET on a cycle ergometer. CPET-related measurements included: oxygen uptake (V’O_2_), carbon dioxide production (V’CO_2_), ventilatory profile, heart rate (HR) and oxygen pulse (V’O_2_/HR) throughout exercise and at lactic threshold (LT) and peak. LT measures represent submaximal exercise related data. PA was assessed using the accelerometer SenseWear Pro3 Armband.

**Results:**

Moderate (>4.8 metabolic equivalents (METS)) and moderate + vigorous (>7.2 METS) PA was related to V’O_2_ (p = 0.005 and p = 0.009, respectively) and work rate (p = 0.004 and p = 0.002, respectively) at LT. Moderate PA or greater was positively related to peak V’O_2_ (p = 0.005 and p = 0.003, respectively). Daily PA levels were similar in CF and healthy controls. Except for peak values, V’O_2_ profile and the V’O_2_ at LT were comparable between CF and healthy controls.

**Conclusions:**

In adult CF patients daily PA positively correlated with aerobic capacity. PA measurements are a valuable tool in the assessment of exercise performance in an adult CF population and could be used for interventional exercise trials to optimize exercise performance and health status. PA levels and parameters obtained at submaximal exercise are similar in CF and in healthy controls.

**Electronic supplementary material:**

The online version of this article (doi:10.1186/s12890-015-0036-9) contains supplementary material, which is available to authorized users.

## Background

In cystic fibrosis (CF) peak oxygen uptake (V’O_2_,_peak_), maximal work rate (Work _max_) and ventilatory equivalents for oxygen uptake (V’E/V’O_2_) measured during incremental cardiopulmonary exercise testing (CPET) are significant predictors of mortality [1,2]. Recently, data obtained in children with CF suggest that aerobic fitness is also associated with lower risk of hospitalization [3]. Although peak exercise tolerance is reduced in CF [4], some studies have shown that aerobic exercise capacity increases with physical training programs [4-6].

The best clinical practice to investigate aerobic fitness includes measurements obtained during CPET, however there are still many difficulties associated with its use in clinical practice [7]. In particular, maximal exercise tests require the subject to make maximal efforts, rendering such tests highly dependent on motivation. Submaximal exercise related data (also known as lactate threshold-LT measures) is an alternative index of aerobic fitness, information that is directly relevant to a patient’s ability to carry out the tasks of daily living and may be useful in the clinical environment in patients who may not be able or willing to provide a maximal effort, especially when maximal exercise tolerance is limited by ventilatory factors [7]. Despite some studies highlighting the use of exercise testing in CF [8,9] and CF guidelines recommending an annual assessment [10], CPET remains underutilised [11]. This is due in part to its expense and the lack of adequately trained staff [11] as well as patient’s dislike of the test itself [9]. So, to investigate this further, different methods of quantifying exercise capacity in CF are required. Modalities that are not excessively stressful to patients are particularly favorable. Field tests, such as shuttle tests, timed walking and step tests are reasonable surrogates for incremental CPET [11,12] and they have the added value of being more reflective of usual physical activity patterns [13]. It has not been investigated in adults with CF whether objective measurements of physical activity (PA) could be added among the CPET surrogates.

Physiological responses to submaximal CPET reflect daily PA at different intensities in a healthy population [14], however no information is currently available in clinically stable adults with CF. To date, the relationship between formal CPET parameters at submaximal workloads and daily activity levels in CF has not been well established. Thus, the possibility that measuring habitual PA by a portable accelerometer could be used to assess the CF aerobic state is worthy of further investigation.

As survival in CF continues to improve a better understanding of the relationship between lifestyle in adulthood and habitual daily PA is important. Few studies have compared daily PA in adult CF patients and healthy subjects. The CF population examined by *Troosters et al.* [15] spent less time in moderately intense PA compared with healthy controls. Conversely, our recent study demonstrated that the time spent in daily PA at different intensities was similar in adults with CF and age-matched healthy controls [16]. Similarly, *Rasekaba et al.* showed that moderate or vigorous habitual PA measured using a validated questionnaire was similar between CF adults and controls [17]. So, it remains unclear whether CF adults have different habitual activities compared with healthy peers and in order to clarify the discrepancies in the current literature further studies are needed. Moreover, despite our knowledge about the beneficial effects of a supervised exercise intervention on submaximal and maximal oxygen uptake in CF [18,19], there have been no studies examining the relationship between habitual daily PA and submaximal fitness in CF.

Therefore the purpose of this study was twofold. First, we aimed to examine the relationship between daily PA and aerobic fitness both at submaximal and maximal levels in clinically stable adults with CF. Specifically, we were interested in evaluating PA as a surrogate of CPET in the assessment of exercise capacity in CF. In addition to this primary aim, we wished to compare both daily PA levels and responses to CPET of adult CF patients and healthy controls. In analyzing the possible positive effect of habitual PA in CF, we sought to evaluate the parameters obtained at submaximal level of exercise in CF and in healthy control peers.

## Methods

### Subjects

Thirty adult CF patients (33 ± 8SD years) with mild to moderate lung disease (FEV_1_ 50%-90% predicted) were recruited consecutively from the CF outpatient clinic at Policlinico Umberto I Hospital (Sapienza University of Rome, Italy). Patients were excluded if they had unstable medical conditions that could cause or contribute to breathlessness (i.e. cardiovascular, metabolic, or other respiratory diseases) or other disorders that could interfere with exercise testing, such as neuromuscular diseases or musculoskeletal problems. Patients were excluded if they had had a pulmonary exacerbation within four weeks of the study period, were on the waiting list for lung transplantation or had undergone lung transplantation.

Fifteen healthy control subjects matched for age were recruited from staff and colleagues of the same hospital by invitation. To be eligible for inclusion, the healthy control subjects needed to have normal baseline spirometry (FEV_1_ ≥ 80%predicted, FEV_1_/FVC ≥0.7) and free of any health problems, including cardiovascular, neuromuscular, musculoskeletal, or respiratory diseases.

### Study design

This was a prospective case–control study. All subjects provided written informed consent. The study was approved by the ethics committee of Policlinico Umberto I Hospital, Sapienza University of Rome, Italy. After consent and appropriate screening of medical history, all subjects underwent pulmonary function testing and performed a symptom-limited incremental CPET. Finally, patients and controls wore an accelerometer (SenseWear Pro3 Armband) to assess daily habitual PA over five consecutive days of their normal activities. Subjects with CF were asked to continue any respiratory-related medications before the visits. All subjects were required to eat a normal mixed diet before laboratory visits to provide valid experimental/metabolic results during the exercise. Subjects were also asked to avoid the ingestion of alcohol, caffeine-containing products, and heavy meals for at least 4 hours, and to refrain from strenuous activity (e.g., cycling, running) for at least 12 hours before testing. Assessment was conducted at the same place and time of day for all subjects.

The calculation for sample size was done using a previous study by *Troosters et al.* [15] who showed a difference in V’O_2_ peak % predicted of 41 with a standard deviation of 13.62. We proposed that our effect size could be about half of that obtained by *Troosters et al.* [15], therefore for 80% power and 5% significance and similar variability it would require a total of 36 subjects in a ratio of 2:1. We therefore recruited 45 in total in a ratio of 2:1.

### Procedures

Spirometry, nitrogen washout and single-breath diffusing capacity (DL_CO_) were performed by standardised techniques using an automated pulmonary function testing system (COSMED PFT, Pavona Italy) [20-22]. All pulmonary function data were standardized as percentages of predicted normal values [23,24].

A maximal incremental exercise test was conducted on an electronically-braked cycle ergometer (COSMED, Pavona Italy) using the Quark b^2^ system (COSMED, Pavona Italy) according to guidelines [25]. CPET consisted of a steady-state resting period, then 2-minutes of warm-up at 10 watt followed by a stepwise protocol in which the work rate was increased in 1-minute intervals by increments of 20 Watt. The test was continued until the point of symptom limitation (peak exercise). Subjects were asked to score their sense of breathlessness and muscle fatigue throughout the exercise and at peak exercise using Borg scale [26]. Oxygen saturation (SpO_2_), by pulse oximetry, electrocardiographic monitoring of heart rate (HR), oxygen uptake (V’O_2_), carbon dioxide production (V’CO_2_) and minute ventilation (V’E) were collected. Exercise parameters were compared with the predicted normal values of *Jones et al.* [27]. Ventilation was compared with the maximal ventilatory capacity (MVC), which was estimated by multiplying the measured FEV_1_ by 40 [28]. The LT was detected individually using the V-slope method [7].

### Total body activity measurement

PA was assessed for five consecutive days. We used the SenseWear Pro3 Armband (BodyMedia Inc., Pittsburgh, PA, USA) which has been validated in CF [29,30]. The sensor contains a biaxial accelerometer (longitudinal and transverse), a galvanic skin response sensor, a heat flux sensor, a skin temperature sensor and a near-body ambient temperature sensor from which the data were stored minute by minute. Using specific software these variables, as well as body weight, height, handedness and smoking status (smoker or non-smoker), were used to estimate the intensity of PA, expressed in metabolic equivalents (METS), total energy expenditure, active energy expenditure (i.e. the number of calories per day due to a physical activity), physical activity duration, time lying down and sleep duration. All subjects positioned the armband on the upper right arm (on the triceps point), as recommended by the manufacturer, and they wore it day and night only removing it for bathing or showering. Patients and healthy controls were asked to continue their normal daily activities and patients were asked to continue any respiratory-related medications. The outputs obtained from the armband was the time spent in PA at different intensities and the definitions for activity levels based on METS were those used by *Troosters et al.* [15]. The time (min) spent with an energy expenditure of 3–4.8 METS was considered “mild” activity (e.g., walking at normal walking speed, carrying out light household work), time spent at 4.8-7.2 METS was considered “moderate” activity (e.g., brisk walking or cycling) and activities with an energy expenditure of >7.2 METS were considered “vigorous” (e.g., running or activity with training effects when applied for a sufficient length of time and at an appropriate training frequency) [31]. Finally, the number of steps was measured.

### Statistical analysis

Categorical data were presented as number and percentage and comparisons done using the *chi-square (χ*^*2*^*) test* or *Fisher’s exact test*. Numeric data were presented as mean ± standard deviation (SD), for normally distributed data and comparisons done using the two sample independent *t-test*, while non-normal numeric data were presented as median (interquartile range, IQR) and comparisons done using the *Mann–Whitney U test.*

Assessment of significant changes in physiological and ventilator parameters (i.e. SpO_2_) from rest to peak of exercise during CPET was performed, within each group of patients, by the use of the *paired samples t-test* and the *Wilcoxon signed rank test* for variables assuming normal and non-normal distribution, respectively.

Since activity monitoring data were not normally distributed the comparison of physical activity data was performed using a Mann–Whitney test in order to investigate the differences between CF patients and healthy controls. These data are reported as median (IQR).

Spearman correlations were utilized to investigate associations such as the relationship between oxygen uptake (at lactic threshold and peak exercise) and PA in the CF group.

To investigate whether exercise capacity was different between CF patients and healthy controls, after correcting for potential confounding covariates like age, BMI, FEV_1_ and gender, the latest square means were computed for the dependent variables using a generalized linear models procedure. These analyses were done for variables that were normally distributed. A pragmatic decision was taken to set the significance level at p ≤ 0.01 to correct for multiple comparisons.

## Results

The study population comprised 30 patients with CF and 15 controls. Baseline characteristics, pulmonary function and daily physical activity data for both groups are shown in Table 1. There were no significant differences in terms of age, sex and body mass index (BMI) between the groups. Of the CF group, 83.3% (n = 25) had pancreatic insufficiency and 43.3% (n = 13) were homozygous for the mutation *F508del* (all remaining patients were compound heterozygous *F508del* with the exception of one patient who had an unidentified second mutation). One patient had CF-related diabetes and all patients had chronic pulmonary infection (*Pseudomonas aeruginosa,* n = 26; *Staphylococcus aureus*, n = 3, *Burkholderia cenocepacia complex*, n = 1)*.* Smoking history was negative for all patients and controls.

**Table 1:** **Anthropometric characteristics, pulmonary function and daily physical activities in patients with cystic fibrosis (CF) and in healthy control subjects**

**Characteristics**	**CF (n = 30)**	**Control (n = 15)**	**p-value**
Male/Female	20/10	10/5	1
Age, yr	33 ± 9	29 ± 5	0.19
BMI, Kg/m^2^	22.5 ± 2.7	23.6 ± 3.1	0.23
**Pulmonary function**			
FEV_1_,L	2.65 ± 0.8	4.51 ± 0.96	<0.0001
FEV_1_, % predicted	71 ± 19	109 ± 11	<0.0001
FEV_1_/FVC, %	68 ± 11	83 ± 3	<0.0001
FVC, L	3.84 ± 1.05	5.35 ± 1.09	<0.0001
FVC, % predicted	86.4 ± 16.7	111 ± 9	<0.0001
TLC, % predicted	96.6 ± 16.3	104 ± 9	0.09
DL_CO_, % predicted	80.2 ± 14	94 ± 11	0.002
MVC, L/min	105.9 ± 32	180.2 ± 38.5	<0.0001
**Daily physical activity**			
Steps, number/day	9160.5 ± 3825.6	10591 ± 4024.6	0.25
Mild intensity activities, min/day	159 (100–246)	147 (77–205)	0.22
Moderate intensity activities, min/day	13 (9–29)	11 (7–16)	0.34
Vigorous intensity activities, min/day	1 (0–3)	1 (0–5)	0.94
Moderate + Vigorous intensity activities, min/day	16 (9–29)	12 (8–27)	0.43

*Definition of abbreviations*: CF = Cystic Fibrosis; BMI = body mass index; FEV_1_ = forced expiratory volume in one second; % pred = % predicted; FVC = forced vital capacity; MVC = maximal ventilator capacity estimated as 40 × FEV_1_; TLC = total lung capacity. Data are presented as mean ± SD or median (interquartile range), unless otherwise stated.

### Daily physical activities

Habitual daily PA of the CF patients and of the age and sex-matched controls included in the study group is shown in Table 1 and in Additional file 1: Table E1 of the online supplement. The number of steps, PA at mild, moderate and vigorous intensity was not different in CF patients compared with healthy control subjects.

### Relationship between daily physical activities and CPET

**Figure 1:**
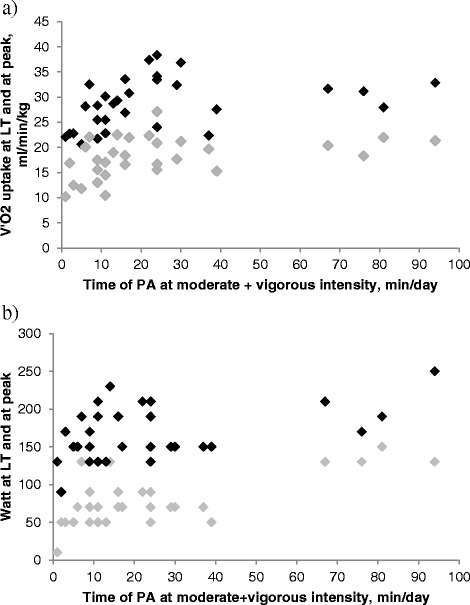
Relationship between the time spent in physical activities (PA) above moderate (4.8-7.2 METS) and vigorous (>7.2 METS) intensity and **a)** oxygen uptake (V’O_2_) at lactic threshold (LT) r = 0.46, *p* = 0.009 and at peak r = 0.51, *p* = 0.003, **b)** watt at lactic threshold (LT) r = 0.52, *p =* 0.002 and peak r = 0.35, *p =* 0.05 in patients with cystic fibrosis*.* □ Values at lactic threshold, ■ values at peak of exercise.

PA of moderate (4.8-7.2 METS) intensity was related to V’O_2_ uptake at LT, expressed as absolute (R = 0.44, p = 0.01) and relative to body weight (R = 0.49, p = 0.005). Physical activities above the threshold of moderate (4.8-7.2 METS) and vigorous (>7.2 METS) intensity were also related to V’O_2_ uptake at LT, expressed as absolute (r = 0.45, p = 0.01) and relative to body weight (r = 0.46, p = 0.009; Figure 1a). There was a relationship between PA and work rate at LT (moderate PA: R = 0.5, p = 0.004; moderate and vigorous PA: R = 0.52, p = 0.002, Figure 1b).

Habitual PA at moderate intensity was positively related to V’O_2,peak_, expressed relative to body weight, (R = 0.49, p = 0.005). Time spent in moderate and vigorous daily activities was correlated to V’O_2,peak_ both expressed as absolute and relative to body weight (R = 0.41, p = 0.02; R = 0.51, p = 0.003, Figure 1a) and to Watt_max_ (R = 0.35, p = 0.05; Figure 1b).

Lung function was not related to the PA outcomes or to the CPET parameters at LT. There was a relationship between FEV_1_ expressed as absolute value and both V’O_2,peak_ and Watt_max_ (R = 0.45, p = 0.01; R = 0.62, p = 0.0002, respectively).

### Physiological responses to CPET

Measurements at the LT and at peak exercise are shown in Table 2. CF patients and controls reached their LT at a similar V’O_2_ and work rate. Patients with CF stopped exercise at a lower peak V’O_2_, work rate and HR than healthy controls. Significant differences at peak exercise remained between the healthy control group and CF patients after correcting for the covariates age, gender, BMI and FEV_1_. Both the CF patients and the healthy control group stopped exercise primarily because of leg discomfort. At the end of exercise, dyspnea and leg discomfort intensity were rated 1.4 and 1.5 Borg units higher in the control group compared with CF patients, respectively.

**Table 2:** **Measurements at the lactic threshold and at peak symptom-limited incremental cycle exercise**

	**LT**			**Peak exercise**		
**Variable**	**CF (n = 30)**	**Control (n = 15)**	**p-value**	**CF (n = 30)**	**Control (n = 15)**	**p-value**
**Dyspnea, Borg scale**	1 ± 1	1.1 ± 1.3	0.8	4.5 ± 2.3	5.9 ± 2.1	0.02
**Leg discomfort, Borg scale**	1.2 ± 1.07	1.3 ± 1.3	0.8	6 ± 1.9	7.5 ± 1.3	0.01
**Work rate, W**	80 ± 35	88 ± 25	0.4	168.6 ± 35.6	214 ± 53	0.001
**% predicted maximum**	41.3 ± 14.9	40 ± 7	0.7	89.03 ± 18.6	97.2 ± 15	0.1
**V’O**_**2**_ **, ml/min**	1186.2 ± 327	1373 ± 306	0.07	1910.8 ± 468.6	2663.2 ± 774	0.001
**% predicted maximum**	47 ± 9	48 ± 7	0.66	76 ± 13	93 ± 16	0.0006
**V’O**_**2**_ **,ml/min/kg**	17.9 ± 4	18.7 ± 2.3	0.48	28.7 ± 5.02	35.9 ± 6	0.0001
**HR, beats∙min** ^**−1**^	118 ± 16	119 ± 15	0.84	154 ± 12	176 ± 10.5	<0.0001
**% predicted maximum**	63.5 ± 9	63 ± 8	0.83	83 ± 8	92 ± 3	<0.0001
**O**_**2**_ **pulse, ml O**_**2**_ **/beat**	10.1 ± 2.5	11.6 ± 2.8	0.06	12.3 ± 2.9	15.1 ± 4.6	0.01
**SpO**_**2**_ **, %**	96 ± 1.8	97.6 ± 1	0.04	94 ± 2.9	97.6 ± 0.8	<0.0001
**V’**_**E**_ **, l/min**	33.3 ± 10.7	31.6 ± 5.5	0.57	69.3 ± 20.9	102.6 ± 30.9	0.0001
**V’**_**E**_ **/MVC, %**	21.9 ± 5.1	19.5 ± 3.1	0.11	45.6 ± 10.5	62.3 ± 13.9	<0.0001

*Definition of abbreviations*: CF = Cystic Fibrosis; HR **=** heart rate; MVC = maximal ventilatory capacity estimated as 40 × FEV_1_; W = work rate; V’O_2_ = oxygen uptake; V’_E_ = maximal minute ventilation. Data are presented as mean ± SD. *p* values are CF versus control within the given stage of exercise.

**Figure 2:**
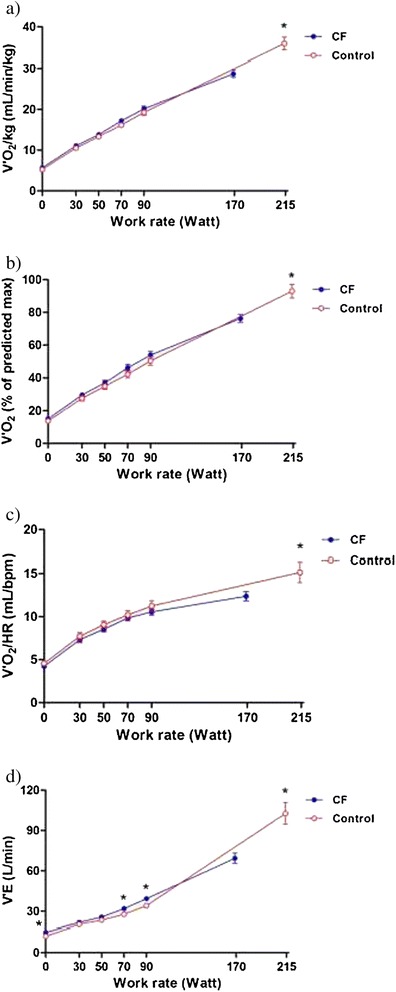
Measurements of incremental cycle exercise. **a**, **b**. Oxygen consumption (V’O_2_). **c**. O_2_ pulse (V’O_2_/HR). **d**. Ventilation (V’E) are shown in response to symptom-limited incremental cycle exercise in patients with cystic fibrosis (CF) and in healthy control subjects. Graphs represent mean ± SE values at rest; at 30, 50, 70, and 90 W during exercise; and at peak exercise. *p ≤ 0.01, CF versus control at a standardized work rate in watts.

The relationships of V’O_2_ versus (vs) work rate as well O_2_ pulse vs work rate were not different between the two groups throughout the exercise (Figure 2a-c). V’E in the CF group was higher at submaximal exercise intensity compared with the healthy group, except at 30 W and 50 W: mean differences in V’E ranged from approximately 4 L/minute at 70 W (p = 0.0003) to 5 L/min at 90 W (p = 0.0003; Figure 2d). The mean peak V’E in CF was less than the predicted MVV with breathing reserve of 31 ± 20%, suggesting that a ventilatory limit was not generally reached. There was no difference in V’E expressed as absolute at the LT between the two groups.

## Discussion

The main finding of this study is that daily PA, particularly of moderate intensity or greater, correlated with measurements of exercise fitness, including V’O_2_ uptake and work rate at LT. Additionally, clinically stable adults with CF and healthy controls have similar daily PA levels and they presented with a V’O_2_ response to CPET and a V’O_2_ at LT comparable to healthy controls.

To our knowledge, this study is the first to investigate the relationship between submaximal exercise data and PA in clinically stable adults with CF and mild to moderate lung disease. The LT is an excellent submaximal index of aerobic performance, information that is directly relevant to a patient’s ability to carry out the tasks of daily living and allows determination of the optimum training intensity in a given individual [7]. We demonstrate that in this clinically stable CF population submaximal CPET indices of aerobic performance are objectively associated with measurements of daily PA. When the relationship between habitual PA and CPET responses was explored in the CF group, there appeared to be significant associations. It was found that PA at moderate and vigorous intensity was closely related to LT parameters, namely V’O_2_ and work rate. Similarly, moderate and vigorous PA was related to VO_2,peak_ and to maximal workloads suggesting that daily PA might play an important role in maintaining aerobic fitness in clinically stable adults with CF. In addition, we postulate that early changes in daily habitual PA may indicate a decline in exercise capacity and therefore may be a sensitive marker of early change in a patient’s disease status. The use of accelerometers has received increasing interest in chronic respiratory diseases [32] and several studies have validated its use in CF [29,30]. So, the possibility of monitoring the CF state of aerobic fitness by measuring habitual physical activity with a portable accelerometer offers obvious advantages.

There are only few studies in which PA levels were objectively assessed and compared with exercise capacity in an adult CF population. The largest study of *Hebestreit et al.* identified that high levels of PA were associated with high aerobic capacity, but the results were obtained recruiting a CF group aged from 12 to 40 years with a mean age of 20 years [33]. *Troosters et al.* confirmed the relationship between PA and VO_2,peak_ in 20 CF patients with a mean age of 25 years [15]. In keeping with that, we confirm in our study the possible role of PA above moderate intensity in the maintenance of better exercise fitness in an older CF population. This is important since it is generally acknowledged that PA at moderate intensity has an important long-term protective effect on health [34] and, as recently shown in CF, prevents muscle strength deterioration after a hospital admission [35].

In this study, the adults with CF were able to accomplish their activities of daily living as much as healthy controls with no differences in terms of PA intensity levels between the two groups. We speculate that these results might follow our clinical practice in terms of patient education to enhance PA levels by explaining all the benefits associated with an active lifestyle. Using the same motion sensor for PA monitoring and the same METS levels, our findings were comparable to those of *Ward et al*. [36] who investigated 24 adults with CF after one month post-discharge, for the mean time spent doing mild and vigorous PA (mild: 159 minutes in our study *vs.* 163 min in Ward’s study; vigorous: 1 min *vs*. 3 min in Ward’s study) despite moderate PA being lower in our study (13 *vs.* 43 min). Moreover, our findings are comparable to those of *Troosters et al.* [15] for the number of steps (9160 *vs.* 9398), times spent in mild and moderate PA (13 minutes in our study *vs*. 14.8 reported by *Troosters et al*.). Vigorous PA was greater in Troosters’s study (1 min *vs.* 4.2). In this latter study the CF population demonstrated step number and PA at mild and vigorous intensity that was not significantly different to healthy subjects, whereas only activities at moderate intensity were reduced in patients with CF [15]. Conversely in our study, we found no differences between CF and controls in any of the PA variables measured. These results could be explained by different PA levels of the healthy control group, especially because our population seems to be more sedentary than *Troosters’s* control group. In fact, despite similar mild PA and number of steps (10591 in our study *vs.* 10281 in Troosters’s study), PA at moderate and vigorous intensity were lower (moderate PA 11 min in our study *vs.* 34 min and vigorous PA 1 min in our study *vs.* 9 min in Troosters’s study). It is possible that differences in PA results may reflect cultural differences, subjects’ motivation to participate in the study, a small sample size or a sampling error. However, with the use of a subjective method (a questionnaire) in a large CF population, new insights into the adult CF patients’ PA habits have been highlighted, showing similarities in moderate and vigorous habitual PA to control groups [17]. Larger studies may be needed to objectively confirm if adults with CF can achieve PA levels similar to the general population.

Our study demonstrated that, despite reduced exercise tolerance, CF patients produced V’O_2_ responses comparable to healthy controls and reached their LT at a work rate and O_2_ uptake similar to that of healthy controls. This is important if we consider that the V’O_2_ at which lactate starts to increase closely reflects the fitness of an individual and can increase with a training program [18]. The novelty of the present study was that we observed these CPET responses in a group of adults with CF who were not engaged in specific home-based exercise training. They were generally active as part of their daily lifestyle, as evidenced by the similar levels of daily PA between CF and healthy controls. The benefits of an exercise program in CF have been largely established by *Cerny et al.* [37] and *Gruber et al.* [18]. The results were obtained during hospitalization for pulmonary exacerbations or during an in-patient rehabilitation course. In both studies, CF patients were under supervision and received several medical interventions (i.e., *i.v*. antibiotics, nutritional supplement, airway clearance therapy, psychological counseling), therefore making it difficult to ascertain which of the above-mentioned factors were responsible for the greatest effect of the observed changes. Moreover, in a previous study, well-nourished female athletes with CF and normal pulmonary function tests reached the LT at a level of exercise intensity equivalent to that of healthy subjects, suggesting similar muscle conditioning [38]. In this sense, the similarity observed in our study between CF and healthy subjects for exercise parameters measured at LT and their correlation with PA in CF population, made us assume that habitual PA may positively affect the submaximal exercise parameters in adult CF.

Finally, our measurements at peak exercise revealed that CF patients’ peak symptom-limited V’O_2_ was reduced by 24% of the predicted normal value and that there were no differences between the CF and the healthy group in the V’O_2_/work rate slopes which were within the normal range. The present study focused on the role of daily physical activity; other factors which may contribute to the reduced exercise tolerance in CF patients were not investigated in detail.

We recognize limitations of the present study. Firstly, this study involved a relatively small number of participants, the majority of which were male, so this limits its generalizability. Secondly, our healthy controls are disease-free but possibly sedentary, even if they were recruited from staff and colleagues of our hospital and they were physiotherapists, students and doctors. Unfortunately, it was technically difficult to recruit a different control group at the time we conducted the study. Further research including a bigger population both for CF patients and for healthy control is needed to highlight potential differences in habitual PA and to better characterize their lifestyle. Finally, CPET variables have been reported to predict survival in CF [1,2] but whether this holds true or not for PA is unknown. Further studies are needed to evaluate the influence of regular PA on this and other important clinical outcomes in CF.

## Conclusions

Daily PA positively correlated with aerobic capacity in adults with CF and mild to moderate lung function impairment and was similar to healthy controls. We have also shown that V’O_2_ profile and the V’O_2_ at LT were comparable between healthy controls and patients with CF. These data suggest daily PA could be considered a valuable tool in the assessment of aerobic fitness in adult CF population. Measuring PA is straightforward and could easily be integrated into interventional exercise trials. Further studies are required to determine if PA measurements are sensitive enough to detect early pathophysiological changes in CF exercise capacity, in order to consider them as a simple and practical method for monitoring the disease status.

## Additional file

Additional file 1: Table E1. Daily physical activities measured by the accelerometer in patients with cystic fibrosis (CF) and control subjects.

## Abbreviations

CF: Cystic Fibrosis
BMI: Body mass index
FEV1: Forced expiratory volume in 1 second
VO_2_: Oxygen uptake
VE: Ventilation
LT: Lactic threshold
