# Supplementary material for: Relationship between daily physical activity and aerobic fitness in adults with cystic fibrosis
Source: BMC Pulm Med. 2015 May 9;15:59. doi: 10.1186/s12890-015-0036-9 (PMC4430900; doi:10.1186/s12890-015-0036-9)
Supplement: Additional file 1: Table E1. — Daily physical activities measured by the accelerometer in patients with cystic fibrosis (CF) and control subjects. [file 12890_2015_36_MOESM1_ESM.docx]

| **Variable** | **CF (n=30)** | **Control (n=15)** | **p-value** |
| --- | --- | --- | --- |
| Total energy expenditure, cal | 2713.3±545 | 2845.8±593.4 | 0.45 |
| Active Energy expenditure, cal | 895.3±525.3 | 749.6±398.1 | 0.34 |
| Duration Physical Activity, min/day | 178 (111-304) | 175 (92-215) | 0.25 |
| Avg mets | 1.77±0.27 | 1.66±0.22 | 0.19 |

**TABLE E1. DAILY PHYSICAL ACTIVITIES MEASURED BY THE ACCELEROMETER IN PATIENTS WITH CYSTIC FIBROSIS (CF) AND CONTROL SUBJECTS**

*Definition of abbreviations*: CF = Cystic Fibrosis; Avg METS: average of metabolic equivalents. Data are presented as mean±SD or median (interquartile range), unless otherwise stated.
